# Development and Validation of Two Analytical Methods Using High‐Performance Liquid Chromatography and Atomic Absorption Spectroscopy for the Analysis of Tunisian Marketed Pharmaceutical Product‐Based Insulin and Zinc: Application in Routine Stability Study

**DOI:** 10.1002/bmc.70585

**Published:** 2026-10-05

**Authors:** Majdi Ben Messaoud, Sarra Mahfoudhi, Dorra Amor, Yassine Mokni, Kaouther Louati, Kaouther Zribi, Asma Omezzine, Fathi Safta

**Affiliations:** ^1^ Faculty of Pharmacy Monastir Laboratory for the Chemical, Galenic and Pharmacology Development of Medicines LR12ES09 Monastir Tunisia; ^2^ Biochemistry Department Sahloul University Hospital Sousse Tunisia; ^3^ Laboratory of Molecular Biology Applied to Neurological and Cardiovascular Diseases, Hereditary Nephropathies and Pharmacogenetics LR12SP11 Sahloul University Hospital Sousse Tunisia; ^4^ Laboratory of Toxicology Hédi Chaker University Hospital Sfax Sfax Tunisia

**Keywords:** A21‐desamido‐insulin, AAS, HPLC, insulin, optimization, stability study, validation, zinc

## Abstract

Given the widespread reliance on insulin among diabetic patients, ensuring its stability is an essential task not only for preserving its therapeutic efficacy but also for safeguarding patient health and safety. In addition, insulin storage practices were frequently misunderstood that may compromise drug potency. Moreover, there were discrepancies in the shelf lives of the different insulin products offered in Tunisia. Therefore, two analytical methods for the analysis of insulin using high‐performance liquid chromatography coupled to ultraviolet detector (HPLC‐UV) and zinc using atomic absorption spectroscopy (AAS) in Tunisian marketed pharmaceutical product‐based insulin were employed. Then the stability of these products was assessed. The study showed good insulin stability at neutral pH exposed to daylight for at least 30 days at 25°C, whereas instability was revealed only in diluted solutions at pH 2. Our work highlights the necessity for a more comprehensive evaluation of insulin stability, particularly under conditions where consistent refrigeration is not feasible. Such an assessment is essential to inform practical storage guidelines and ensure therapeutic efficacy in resource‐limited settings.

## 1 Introduction

Insulin is a polypeptide hormone synthesized by Langerhans beta cells (Mayer et al. 2007). This later plays a critical role in homeostasis by regulating blood sugar and through its metabolic effects on lipids and proteins. Insulin is recommended not only in diabetes‐type 1 but also in some cases of diabetes‐type 2. After its discovery in 1921 (Scheen and Lefèbvre 2021) and due to the lack of knowledge of its chemical structure, in vivo bioassays have been developed to assess its activity through inauguration of hypoglycemia in rabbits and convulsions in mice (Banting and Best 1987). After which, the establishment of its chemical structure has allowed the development of several analytical methods for its analysis and quality controls, namely, enzyme immunoassay, electro‐chemiluminescence, radio‐immunology, capillary electrophoresis, and high‐performance liquid chromatography either coupled to mass‐spectrometry (HPLC‐MS) or ultraviolet detector (HPLC‐UV) (Shen et al. 2019).

Insulin is usually associated with zinc since this latter plays a crucial role in insulin biosynthesis, storage, and secretion. In the field of chemistry, six insulin molecules are associated with two atoms of zinc to form a hexamer, which represents the inactive storage form that must be converted to an insulin monomer to achieve its biological activity. Insulin hexamer is the most stable form compared to the insulin dimer and monomer (Dunn 2005).

Currently, the differently marketed human insulin products (insulin soluble, insulin isophane, and insulin biphasic isophane) contain zinc (European Pharmacopoeia 2023). Total zinc should not exceed 40.0 μg per mL of insulin solution or suspension (100 IU/mL or 3.47 mg/mL) (European Pharmacopoeia 2023). Screening for trace elements in pharmaceutical products, such as zinc, is usually a challenging task due to their existence at very low concentrations. Following the literature, several analytical methods have been described for zinc determination, including atomic absorption spectroscopy (AAS), titration, colorimetric method, ion chromatography, HPLC coupled to refractive index detector, inductively coupled plasma optical emission spectrometer (ICP‐OES), inductively coupled plasma mass spectrometer (ICP‐MS), and X‐ray fluorescence spectroscopy (Vanga et al. 2024; Hall and King 2022; Marukhlenko et al. 2021; Shvetsova et al. 2023; Song et al. 1976). The ICP‐MS and ICP‐OES methods are gaining more popularity, but AAS is still used for its simplicity and cost‐effectiveness (Shkunnikova et al. 2021).

Insulin stability remains the most crucial aspect to ensure drug‐efficacy and patient safety in diabetes therapy management. However, various factors, including formulation, production, and storage conditions, could influence the stability of insulin products (Heinemann et al. 2021).

The storage conditions of insulin are frequently misunderstood. By way of illustration, biological products differed in terms of shelf life after opening. In addition, some manufacturers did not provide adequate data to correctly evaluate feasible storage conditions, especially in specific hot tropical climates, rural areas, and conflict situations with limited access to refrigeration (Heinemann et al. 2021; Kaufmann and Boulle 2021).

Therefore, the aim of this study was to develop and validate a simple and rapid analytical method using HPLC‐UV to simultaneously separate and analyze insulin from its excipients and the main degradation product (A21‐desamido‐insulin). Then, we validated another method by AAS for the assay of zinc in insulin products. The advantages and limits of both methods will be discussed. Furthermore, we applied our developed HPLC‐UV method to study the effects of pH with daylight exposure on insulin stability and shelf life, in some Tunisian marketed product‐based human insulin. Similarly, we applied the AAS method to study the influence of daylight exposure on zinc content in the tested insulin products.

Following our knowledge, our investigation could be new, since no study was reported yet for the simultaneous analysis of both insulin and zinc in insulin products. Strikingly, our study is endowed with great interest in the field of the pharmaceutical industry, since the assessment of these two components is fundamental as required by the European pharmacopoeia (2023) and the USP (2024) in order to ensure the quality of such products and hence patient safety.

## 2 Materials and Methods

### 2.1 Chemicals and Reagents

For this work, we adopted the following reagents: HPLC‐grade acetonitrile (HONEYWELL), HPLC‐grade methanol (HONEYWELL), hydrochloric acid 37% (PANREAC APPLICHEM), orthophosphoric acid (SIGMA‐ALDRICH), nitric acid 69% (SIGMA‐ALDRICH), phenol (INVITROGEN), M‐cresol (MERCK), glycerol (OXFORD LA), zinc sulfate heptahydrate 99% (znSO4, 7H2O) (LOBACHEMIE), and anhydrous disodium hydrogen phosphate (VWRCHEMICALS).

#### 2.1.1 Standards

Standard insulin was donated from a local pharmaceutical company, and standard zinc stock solution 1 g/L was obtained from PERKIN ELMER for AAS calibration.

#### 2.1.2 Insulin Products

Human insulin solutions or suspensions for injection were purchased from a local pharmacy. Three types of human insulin produced by two different manufacturers (A and B) were studied: insulin soluble, insulin isophane, and insulin isophane biphasic.

### 2.2 Instrumentation

#### 2.2.1 Instrumentation and Chromatographic Parameters for the Assay of Insulin

For the separation and quantification of insulin, we used an Agilent 1260 HPLC system, equipped with a G7111A quaternary pump, a G7129A autosampler, a G7114A variable wavelength detector and Agilent OpenLAB CDS ChemStation software (Rev C.01.07). The chromatographic separation was performed at 25°C using thermo scientific ODS Hypersil column (200 * 4.6 mm) packed with 5‐μm diameter particles. The mobile phase consisted of 28% acetonitrile, 12% methanol, and 60% of 0.2 M of phosphate buffer pH 3 adjusted by phosphoric acid (V/V/V), filtered through a 0.22‐μm membrane filter and degassed prior to use. The detection wavelength and the flow rate were 214 nm and 1 mL/min, respectively. The injection volume was 20 μL.

#### 2.2.2 Instrumentation for the Assay of Zinc

For the quantification of zinc, we used a flame AAS Perkin Elmer Pinaacle 500, which is equipped with a stainless steel nebulizer, an autosampler S23 (Perkin Elmer), a mono‐elemental zinc hollow cathode lamp (Lumina), and Syngistix Touch software. The detection wavelength by this lamp was set at 213.9 nm, corresponding to the zinc maximum absorbance. The air flow rate was set at 10 L/min, and the acetylene flow rate was 2.5 L/min. We used an acetylene tank and an air compressor to generate a gas mixture to fuel the flame. Also, we used a 2% nitric acid solution to wash the automatic injector between two successive analyses (European Pharmacopoeia 2023; USP 2024). The AAS was qualified and calibrated before use.

### 2.3 Preparation of the Solutions

#### 2.3.1 Preparation of the Insulin Solutions

##### 2.3.1.1 Preparation of Standard Insulin Solutions for the Calibration Curve

Insulin standard powder was dissolved in 0.01M HCl to obtain concentrations of 0.6, 0.8, 1, 1.2, and 1.4 mg/mL to be injected immediately in the HPLC column.

##### 2.3.1.2 Preparation of Standard Insulin Solutions Within the Matrix for the Calibration Curve

We prepared other solutions as the previous insulin standard solutions by adding the following excipients: glycerol, phenol, and M‐Cresol in order to obtain concentrations of 16, 2, and 2.5 mg/mL, respectively (Fisher 1955).

##### 2.3.1.3 Preparation of A‐21 Desamido‐Insulin

Insulin standard powder was dissolved in HCl 0.01M to obtain a concentration of 0.2 mg/mL. This solution was transferred to a clean test tube and incubated at 37°C for 28 days to degrade approximately 90% of insulin into A21‐desamido‐insulin.

##### 2.3.1.4 Sample Preparation

The products containing human insulin at a concentration of 3.47 mg/mL and excipients (glycerol, phenol, M‐cresol) were diluted immediately just before analysis with HCl 0.01 M in order to obtain a concentration of 1 mg/mL of insulin.

#### 2.3.2 Preparation of Zinc Solution

##### 2.3.2.1 Preparation of Standard Zinc Solutions for the Calibration Curve

ZnSO_4_ standard powder was dissolved in 0.01M HCl to obtain zinc concentrations of 0.2, 0.6, 1, 1.2, and 1.6 μg/mL.

##### 2.3.2.2 Preparation of Standard Zinc Solutions Within the Matrix for the Calibration Curve

We prepared other solutions as the previous standard solutions by adding the following excipients: glycerol, phenol, and M‐Cresol in order to obtain concentrations of 16, 2, and 2.5 mg/mL, respectively (Fisher 1955).

##### 2.3.2.3 Sample Preparation

The products of human insulin were diluted with HCl 0.01M (1/40) just before analysis.

### 2.4 Validation of HPLC and AAS Methods

The validation of an analytical method was assessed according to the ICH guidelines Q2 (R2) (International Council for Harmonisation 2023), which contains the following tests: specificity, accuracy, precision, matrix effect, calibration curve and range, robustness, and the determination of the limit of detection (LOD) and the limit of quantification (LOQ).

#### 2.4.1 Specificity

The specificity of the insulin assay method was assessed by comparing the chromatograms obtained, respectively, from the blank solution (HCl 0.01M), the matrix solution consisting of a mixture of excipients (M‐cresol and phenol), the standard solution of insulin, and the product solution based on the insulin and matrix mixture spiked with A21‐desamido‐insulin. Peak purity was also checked by assessing the match factor for insulin and A21‐desamido‐insulin peaks.

The specificity of the zinc determination method was assessed by comparing the absorbance of six standard zinc solutions at 1 μg/mL and six other standard zinc solutions at 1 μg/mL spiked with the matrix. The statistical test used was the Mann–Whitney *U* test.

#### 2.4.2 Linearity

Following some previous assays of optimization, the linearity range was found to be between 0.6 and 1.4 mg/mL for the assay of insulin and between 0.2 and 1.6 μg/mL for the assay of zinc. Hence, in order to validate these ranges for each method, three sets of standard solutions were freshly prepared at five different concentrations and three other sets of standard solutions based on the matrix were also freshly prepared at five different concentrations to be analyzed each day during 3 days. Linearity was tested by calculating the coefficient of determination *r* ^2^ that should be superior to 0.990.

Moreover, we applied the following statistical tests for each obtained curve: comparison of the intercept with 0 (Student's T‐test), existence of a significant slope (Fisher *F*1), intragroup variance homogeneity (Cochran *C*), and validity of the regression (Fisher *F*2).

Then, we compared the curves obtained from the standard solutions with those obtained from the matrix solutions, respectively, for the two methods: comparison of the intercepts (Student's T‐test) and the slopes of the regression curves (Student's T‐test).

All calculated statistical tests should be inferior to the corresponding tabulated values except for the Fisher *F*1 test.

#### 2.4.3 Precision

The intraday precision (CVr: coefficient of variation of repeatability) and interday precision (CVR: coefficient of variation of reproducibility) were assessed by analyzing the drug solution in six injections on the same day and on consecutive days, respectively. In our case, a total of 18 different standard solutions spiked with the matrix at the middle‐range concentration for each method were analyzed. The variance homogeneity test (Cochran) was calculated for both methods. CV inferior to 2% was the accepted limit, and the calculated Cochran test should be inferior to the tabulated value.

#### 2.4.4 Accuracy

The study of accuracy was coupled to the linearity by fortifying the solutions with known concentrations of the corresponding drug. The percent recovery of the added drug was determined for both methods. In addition, some statistical tests were evaluated, namely, the intragroup variance homogeneity (Cochran), the validity of average (Fisher), and the average recovery with its 95% confidence interval (Student table). The calculated Cochran and Fisher tests should be inferior to the tabulated values. Average recovery should be close to 100% and included within the 95% confidence interval.

#### 2.4.5 Robustness

Robustness was performed using a design of experiment (DoE) approach under varying chromatographic conditions in and around the optimized parameters (ICH Q14 2023).

The design was Box–Behnken 3 factors with three levels. Three variables were chosen as critical analytical parameters including ACN%, buffer concentration, and flow rate. Variation of ACN% was approximately ± 1.8%. Variation of buffer concentration and flow rate was ± 10%.

The critical analytical attributes were insulin peak symmetry (As), resolution between insulin and A21‐desamido‐insulin (R1), and resolution between A21‐desamido‐insulin and phenol (R2).

#### 2.4.6 LOD and LOQ

For the assay of insulin, the LOD and the LOQ were based on signal‐to‐noise. A signal‐to‐noise ratio of 3:1 and a signal‐to‐noise ratio of 10:1 are generally considered acceptable for estimating the LOD and LOQ, respectively.

For the assay of zinc, the LOD and the LOQ were based on the standard deviation of the blank and the slope. The LOD and the LOQ could be expressed as follows:

$$\mathrm{LOD}=\frac{3.3\times \sigma }{S}\;\text{and}\;\mathrm{LOQ}=\frac{10\times \sigma }{S}$$

*S* is the slope of the regression line, and 𝜎 estimation is based on the standard deviation of the blank.

### 2.5 Stability

#### 2.5.1 Insulin Stability

Following the ICH Q5C for stability testing of biotechnological/biological products, we have conducted some stress degradation study of the insulin products in order to check their quality over time and hence define the practical stability of insulin products: Insulin products were exposed to daylight at 25°C, and samples issued from each product were analyzed three times immediately after opening the vials, then after 10, 20, and 30 days. Each sample was diluted to have the final concentration of insulin at 1 mg/mL in HCl 0.01 M prior to the chromatographic injection.

To assess the influence of low pH on the stability of insulin products, samples issued from each product were diluted to a final concentration of insulin at 1 mg/mL in HCl 0.01 M and then kept diluted at pH 2 before exposure to daylight at 25°C. After which, they were analyzed after 10, 20, and 30 days. Each sample was analyzed three times.

#### 2.5.2 Zinc Stability

To assess the stability of zinc in insulin products exposed to daylight at 25°C, solutions issued from each product were analyzed five times immediately after opening the vials and after 30 days. Then, the determined zinc assays were compared between the two different times using the Mann–Whitney statistical test.

## 3 Results

### 3.1 Insulin Assay Method

#### 3.1.1 Development of an Analytical Method for the Assay of Insulin

Some preliminary experiments had been carried out starting with a C18 column (200 mm * 4.6mm, 5‐μm particle size). The mobile phase consisted of a mixture of phosphate buffer 0.1M at pH 3, acetonitrile, and methanol, used, respectively, at 62%, 26%, and 12% (V/V/V) in an isocratic mode. The flow rate was 1 mL/min, and the injection volume was 20 μL. The detection wavelength was set at 214 nm. These chromatographic conditions were selected based on the study of Najjar et al. (2014). The tested solution was composed of insulin 1 mg/mL in HCl 0.01M within the matrix that is a mixture of the following excipients: glycerol, phenol, and M‐cresol, spiked with A21‐desamido‐insulin.

By adopting these analytical parameters, the resulted insulin peak was short and broad with tailing. The retention time (RT) was 21.4 min, with no separation between insulin and its major degradation product, A21‐desamido‐insulin, as shown in Figure 1.

**FIGURE 1:**
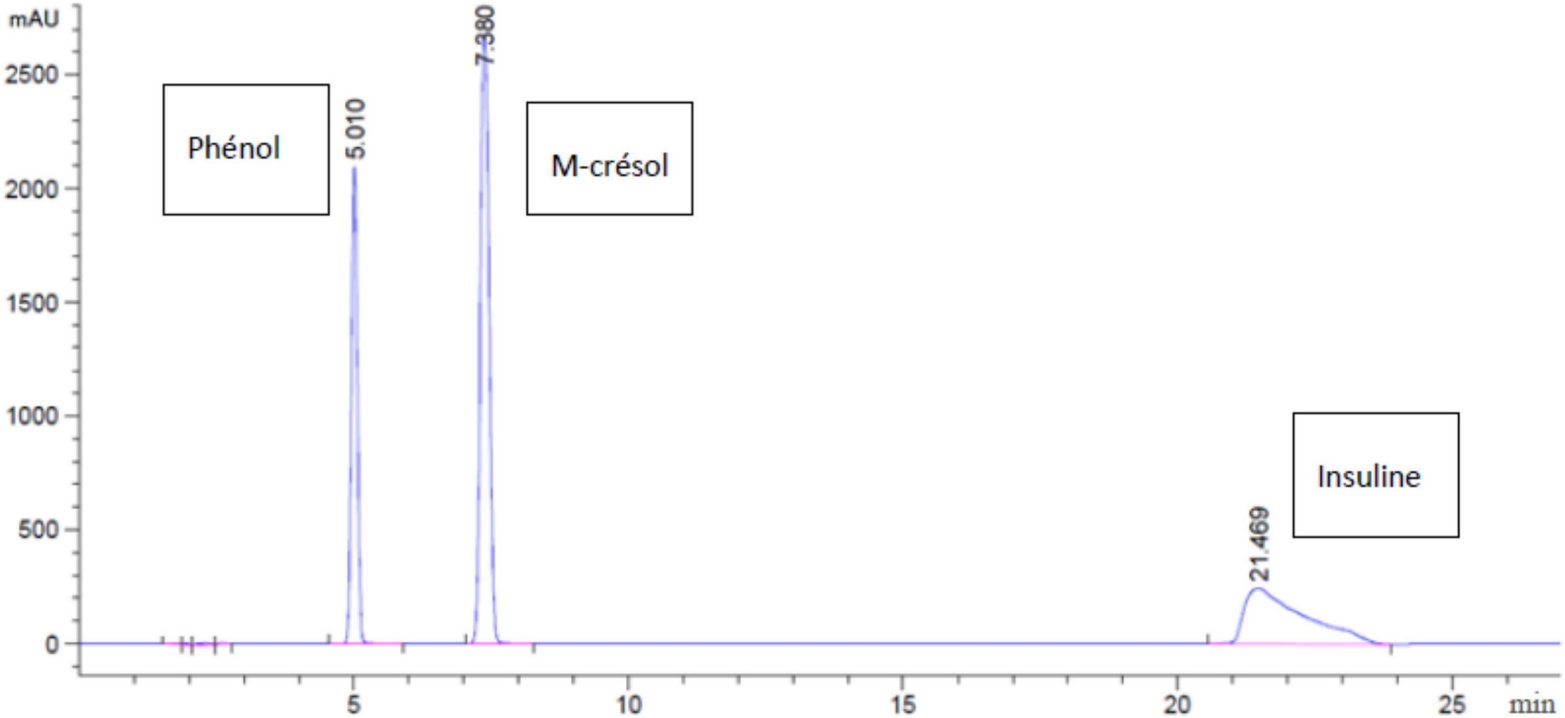
Chromatogram of insulin spiked with A21‐desamido‐insulin in the matrix: The mobile phase consisted of a mixture of phosphate buffer 0.1 M at pH 3, acetonitrile, and methanol (62%, 26%, and 12%).

From this drawback, we first tried to optimize the acetonitrile proportion in order to reduce the RT of insulin and improve peak symmetry as well as the resolution between closed peaks. Hence, we had increased acetonitrile proportion to 38%, while maintaining methanol at 12%. As a result, the insulin was eluted before the phenol and M‐cresol excipients at RT of 2.2 min. Its relative peak was sharp but with tailing, which corresponded to the A21‐desamido‐insulin co‐eluted with insulin (as shown in Figure 2). After which, by reducing the percentage of acetonitrile to 30% in the mobile phase and using 12% of methanol and 58% of phosphate buffer 0.1M pH at 3, the two peaks corresponding to insulin and its degradation product were overlapped as demonstrated in Figure 3. Then, after decreasing acetonitrile to 28% and maintaining the percentage of methanol at 12%, we obtained a small peak between Insulin and phenol, corresponding to A21‐desamido‐insulin (as presented in Figure 4).

**FIGURE 2:**
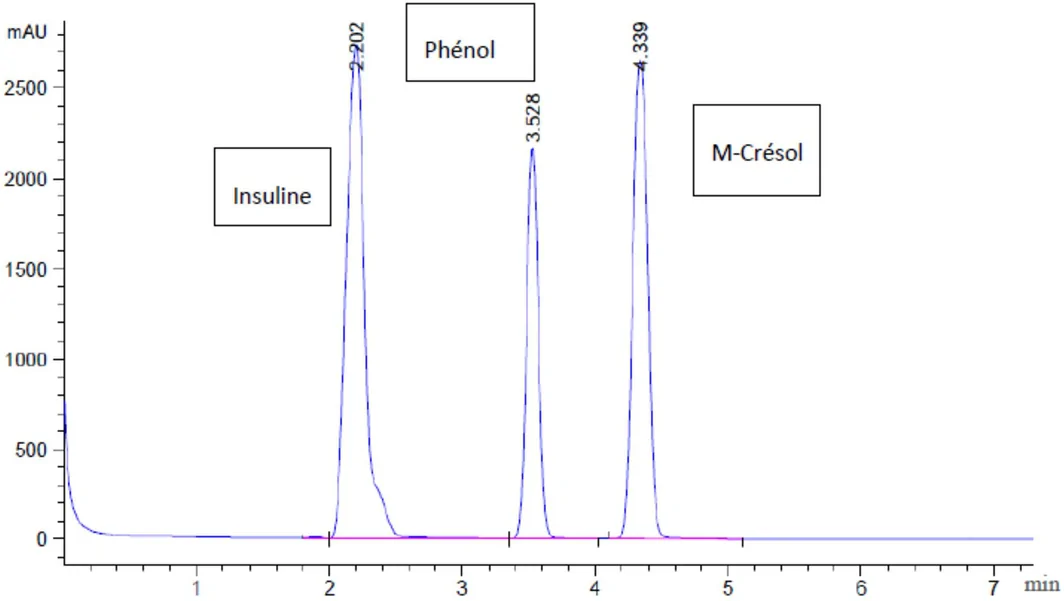
Chromatogram of insulin spiked with A21‐desamido‐insulin in the matrix: The mobile phase consisted of a mixture of phosphate buffer 0.1 M at pH 3, acetonitrile, and methanol (50%, 38%, and 12%).

**FIGURE 3:**
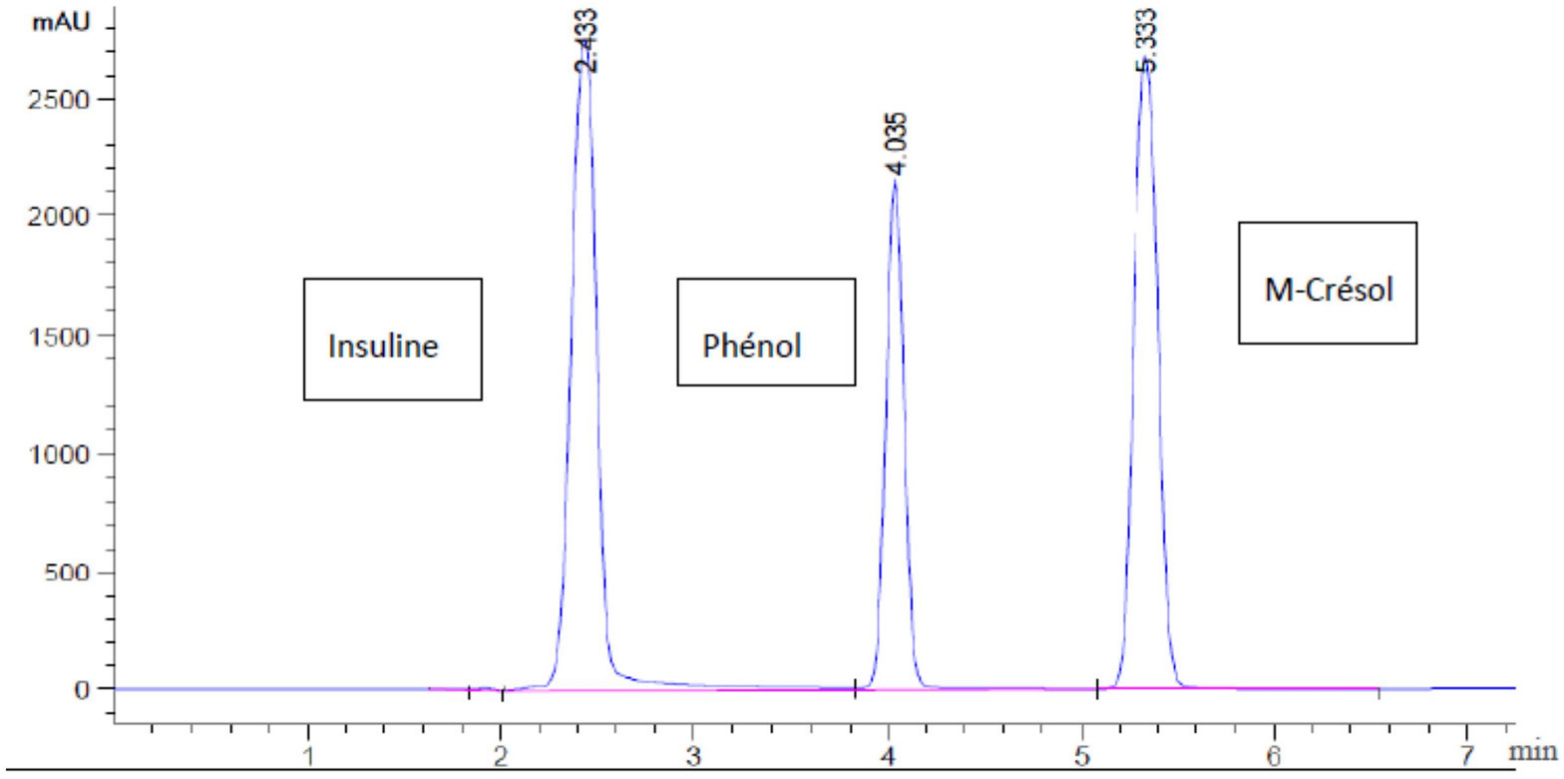
Chromatogram of insulin spiked with A21‐desamido‐insulin in the matrix: The mobile phase consisted of a mixture of phosphate buffer 0.1 M at pH 3, acetonitrile, and methanol (58%, 30%, and 12%).

**FIGURE 4:**
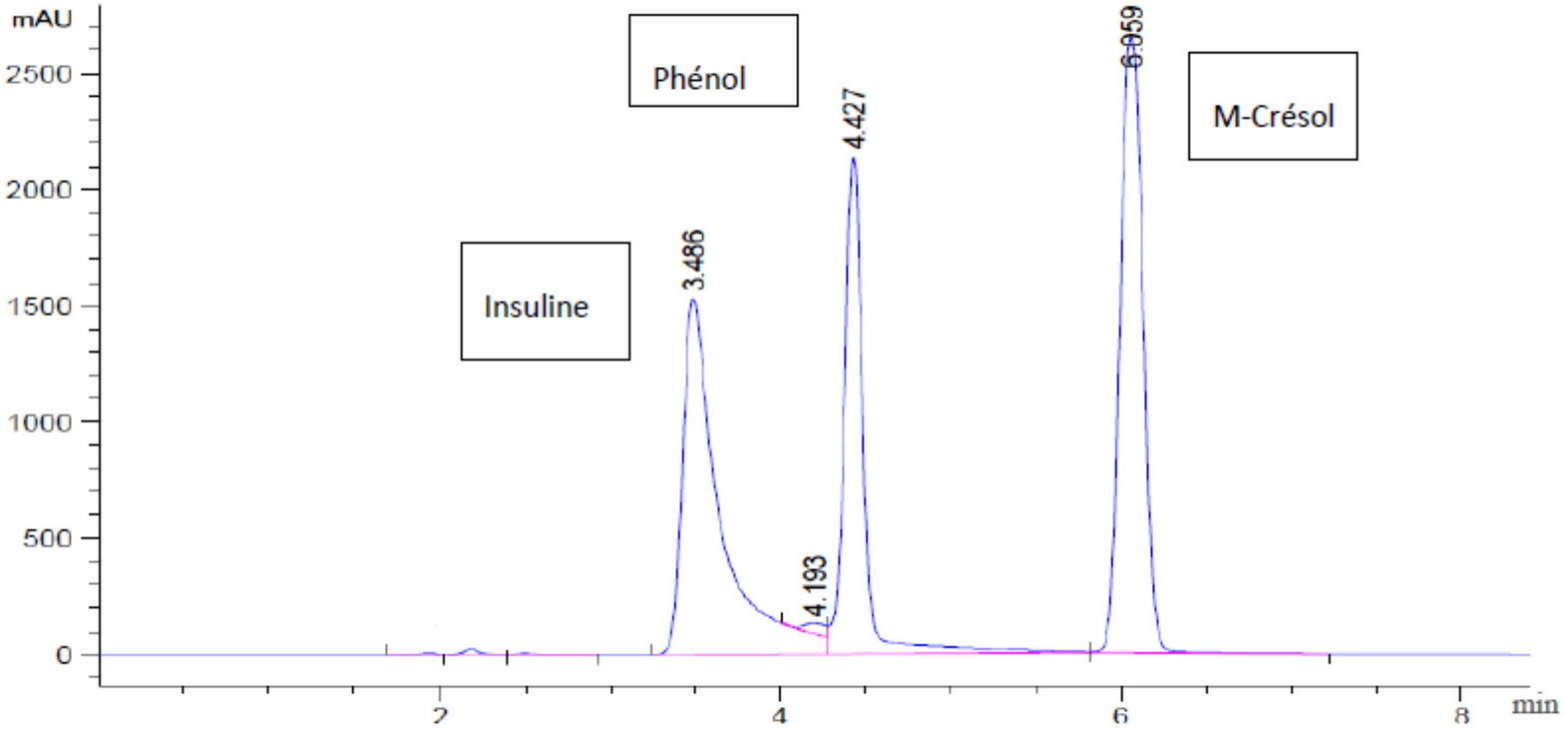
Chromatogram of insulin spiked with A21‐desamido‐insulin in the matrix: The mobile phase consisted of a mixture of phosphate buffer 0.1 M at pH 3, acetonitrile, and methanol (60%, 28%, and 12%).

Secondly, we tried to optimize the concentration of the phosphate buffer as follows: We changed the concentration of phosphate buffer from 0.1 to 0.2 M while maintaining the previous composition of the mobile phase. As a result, we obtained good separations, respectively, between insulin and A21‐desamido‐insulin with a resolution (R) of 1.74 and between A21‐desamido‐insulin and Phenol with R of 2.25 (as shown in Figure 5). Also, the peaks' shape of insulin and A21‐desamido‐insulin were acceptable with symmetry factors between 1.7 and 1.8. Third, we tried to optimize the flow rate by increasing it with 0.1mL/min level from 1 to 1.4 mL/min. As a result, the RT of all the components were reduced, but no improvement in the resolution between insulin and A21‐desamido‐Insulin was noticed. So we maintained the flow rate at 1mL/min.

**FIGURE 5:**
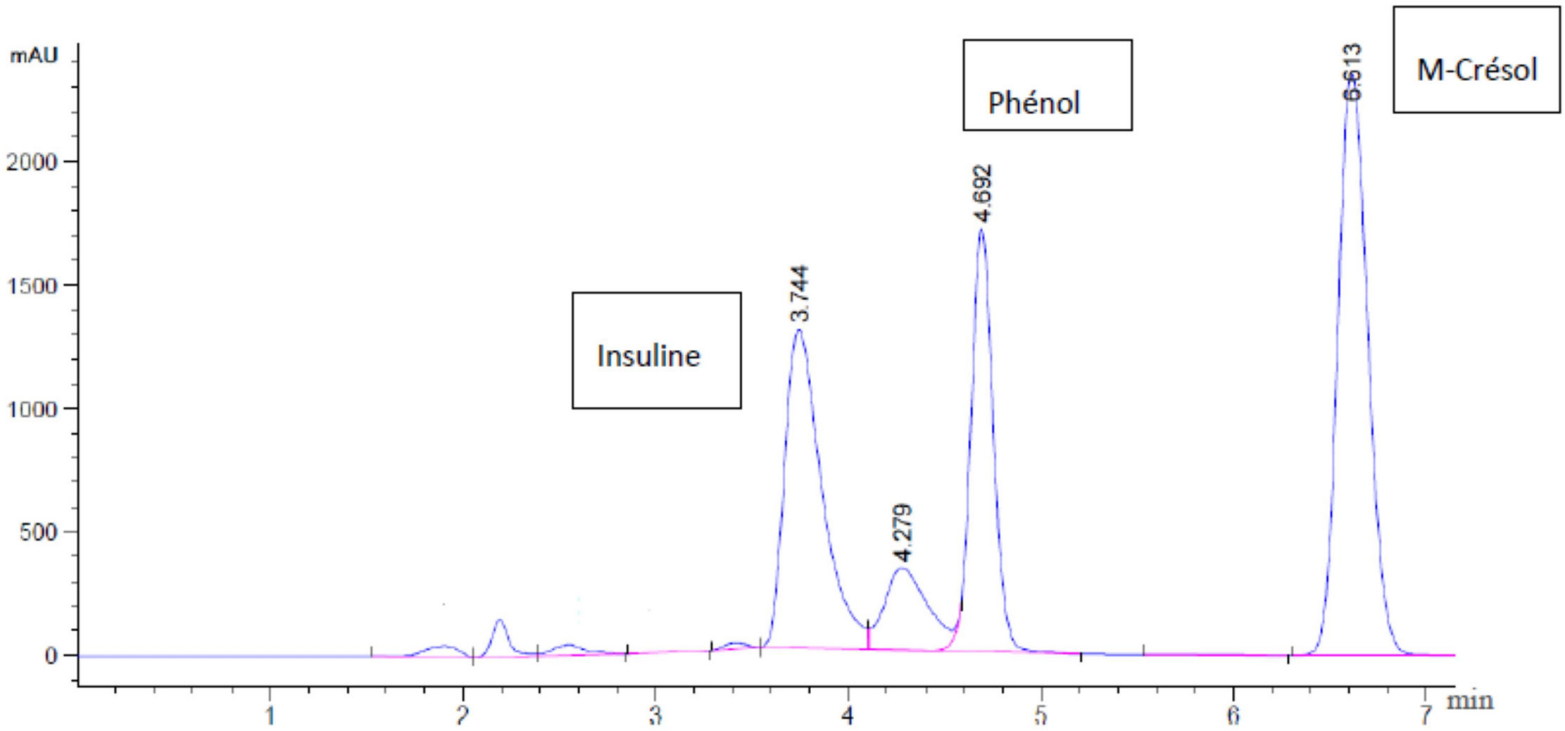
Chromatogram of insulin spiked with A21‐desamido‐insulin in the matrix: The mobile phase consisted of a mixture of phosphate buffer 0.2 M at pH 3, acetonitrile, and methanol (60%, 28%, and 12%).

At the same time, we developed another method by using a gradient mode for both the mobile phase and the flow rate. Several preliminary experiments had been carried out to finally retain a gradient mode for the mobile phase as follows: Acetonitrile increased from 25% to 28%, while phosphate buffer at 0.2 M, pH 3 decreased from 63% to 60% in 4 min, and methanol was fixed at 12%. The flow rate gradient was increased from 1.2 to 1.6 mL/min in 4 min.

As a final result, the insulin and its degradation product were eluted after the phenol and M‐cresol excipients, respectively, at 5.6 min and 6.1 min (as shown in Figure 6). The peaks' shape of insulin and A21‐desamido‐insulin were good with symmetry factors between 1.5 and 1.6. The resolution was also improved, respectively, between insulin and A21‐desamido‐insulin (*R* = 2.55) and between insulin and M‐cresol (*R* = 3.23).

**FIGURE 6:**
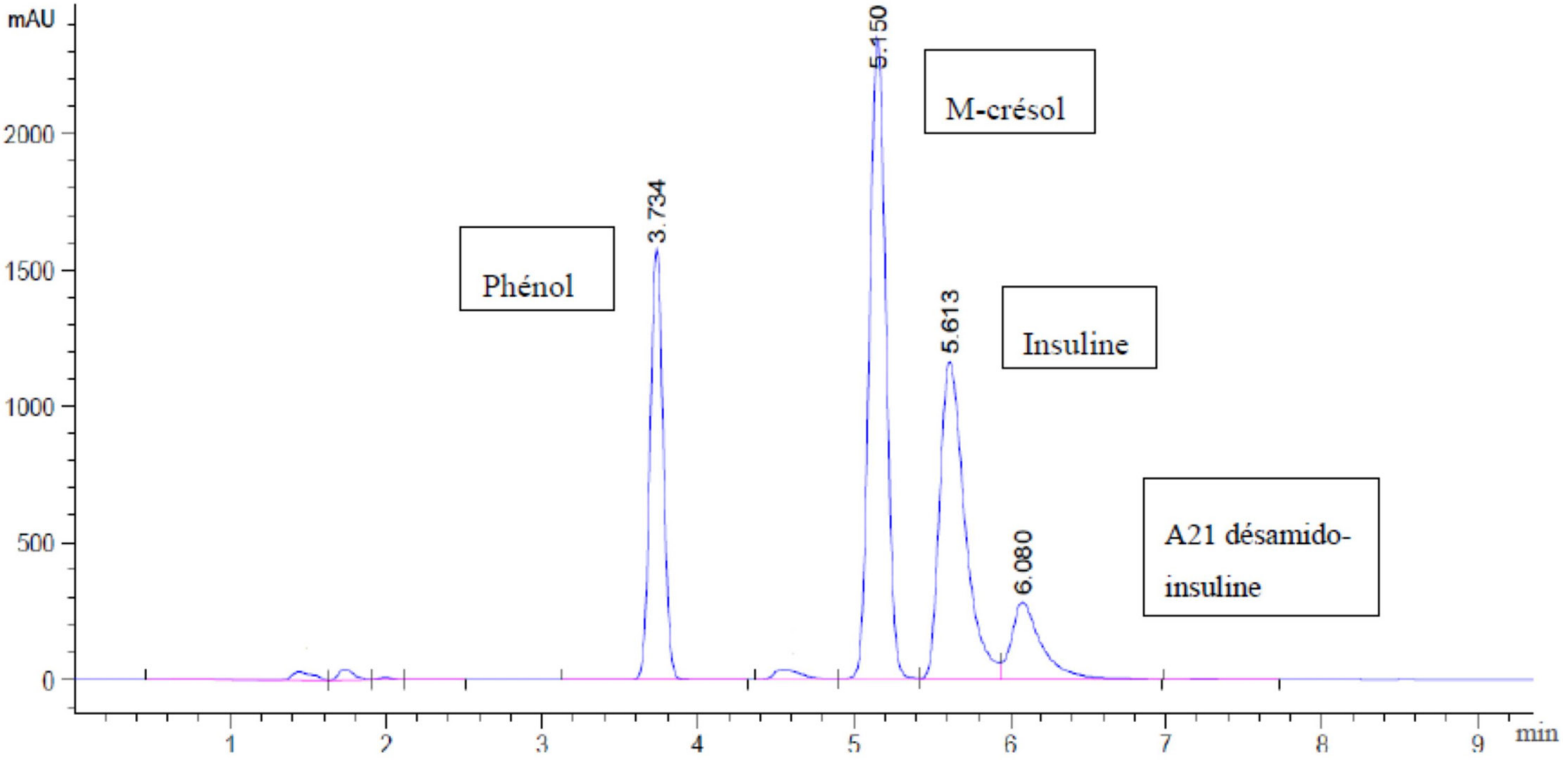
Chromatogram of insulin spiked with A21‐desamido‐insulin in the matrix: using a gradient mode for the mobile phase (Acetonitrile from 25% to 28% in 4 min + fixed 12% methanol) and a linear gradient flow (from 1.2 to 1.6 mL/min in 4 min).

#### 3.1.2 Validation of the Developed Method by HPLC

Only the isocratic method was validated according to the following criteria.

##### 3.1.2.1 Specificity

There was no interference between peaks of insulin, A21‐desamido‐insulin, and excipients (phenol and M‐cresol). The glycerol peak did not appear in the chromatograms since it does not absorb at 214 nm. The match factor was 1000 for both insulin and A21‐desamido‐insulin peaks proving no co‐eluting substances.

##### 3.1.2.2 Linearity

Two calibration curves D1 and D2 were established, respectively, for the insulin standard solution and the insulin standard solution within the matrix. Our results revealed a good linear relationship in the range of 0.6–1.4 mg/mL. The coefficient of determination *R* ^2^ was 0.998 for both curves. The regression equations for the calibration curves were *Y* = 18573x‐106 and *Y* = 18366x + 72.53 for D1 and D2, respectively. There was no significant difference in terms of intercepts (calculated *T*‐value *T*_cal_: 0.35 < tabulated *T‐*value *T*_tab_(0.05; 26): 2.056) and slopes (*T*_cal_: 0.42 < *T*_tab_(0.05; 26): 2.056) between D1 and D2.

The linearity statistical tests are summarized in Table 1.

**TABLE 1:** Results of the statistical tests for the linearity study (insulin assay method).

Test	Tabulated value	Calculated value for D1 (insulin without matrix)	Calculated value for D2 (insulin within matrix)
Homogeneity of the variance: Cochran (C)	*C*_tab_(0.05; 5; 2): 0.68	0.56	0.40
Existence of a significant slope: Fischer (*F*1)	*F*1_tab_(0.05; 1; 13): 4.67	2966.33	2826.92
Validity of the regression line (*F*2)	*F*2_tab_(0.05; 3;10): 3.71	2.59	2.08
Comparison of the intercept with 0	*T*_tab_(0.05; 13): 2.16	0.29	0.20

##### 3.1.2.3 Precision

The coefficients of intraday and interday variations were, respectively, 0.61% and 0.63%. The variance homogeneity Cochran test passed with calculated *C*‐value C_cal_ 0.550 < tabulated *C*‐value C_tab_(0.05; 3; 6): 0.677.

##### 3.1.2.4 Accuracy

The average recovery was 99.86%, and its interval of confidence was between 98.68% and 101.04%.

The intragroup variance homogeneity Cochran test passed with calculated *C‐*value *C*_cal_ 0.067 < tabulated *C‐*value *C*_tab_(0.05; 5; 2): 0.68. The validity of average Fisher test passed with calculated *F*‐value *F*_cal_ 2.53 < tabulated *F*‐value *F*_tab_(0.05; 4; 10): 3.48.

##### 3.1.2.5 Robustness

The Box–Behnken DoE results are summarized in Table 2. Insulin peak symmetry and both resolutions R1 and R2 were acceptable in all experiments proving the robustness of the developed method. Figure 7 illustrates the DoE surface plots.

**TABLE 2:** Box–Behnken DoE results.

OrdreStd	OrdEssai	Flow rate	ACN%	Buffer concentration	R1	R2	As
14	1	1	28	0.2	1.75	2.24	1.72
6	2	1.1	28	0.18	1.68	2.18	1.72
2	3	1.1	27.5	0.2	1.73	1.56	1.78
15	4	1	28	0.2	1.76	2.28	1.69
5	5	0.9	28	0.18	1.65	2.11	1.71
7	6	0.9	28	0.22	1.69	2.22	1.71
1	7	0.9	27.5	0.2	1.71	1.54	1.77
9	8	1	27.5	0.18	1.75	1.58	1.79
11	9	1	27.5	0.22	1.76	1.56	1.77
13	10	1	28	0.2	1.75	2.25	1.71
12	11	1	28.5	0.22	1.59	2.37	1.71
8	12	1.1	28	0.22	1.71	2.19	1.7
4	13	1.1	28.5	0.2	1.63	2.29	1.65
3	14	0.9	28.5	0.2	1.62	2.33	1.68
10	15	1	28.5	0.18	1.55	2.35	1.72

*Note:* R1: Resolution between insulin and A21‐desamido‐insulin. R2: Resolution between A21‐desamido‐insulin and phenol. As: Symmetry factor of insulin peak.

**FIGURE 7:**
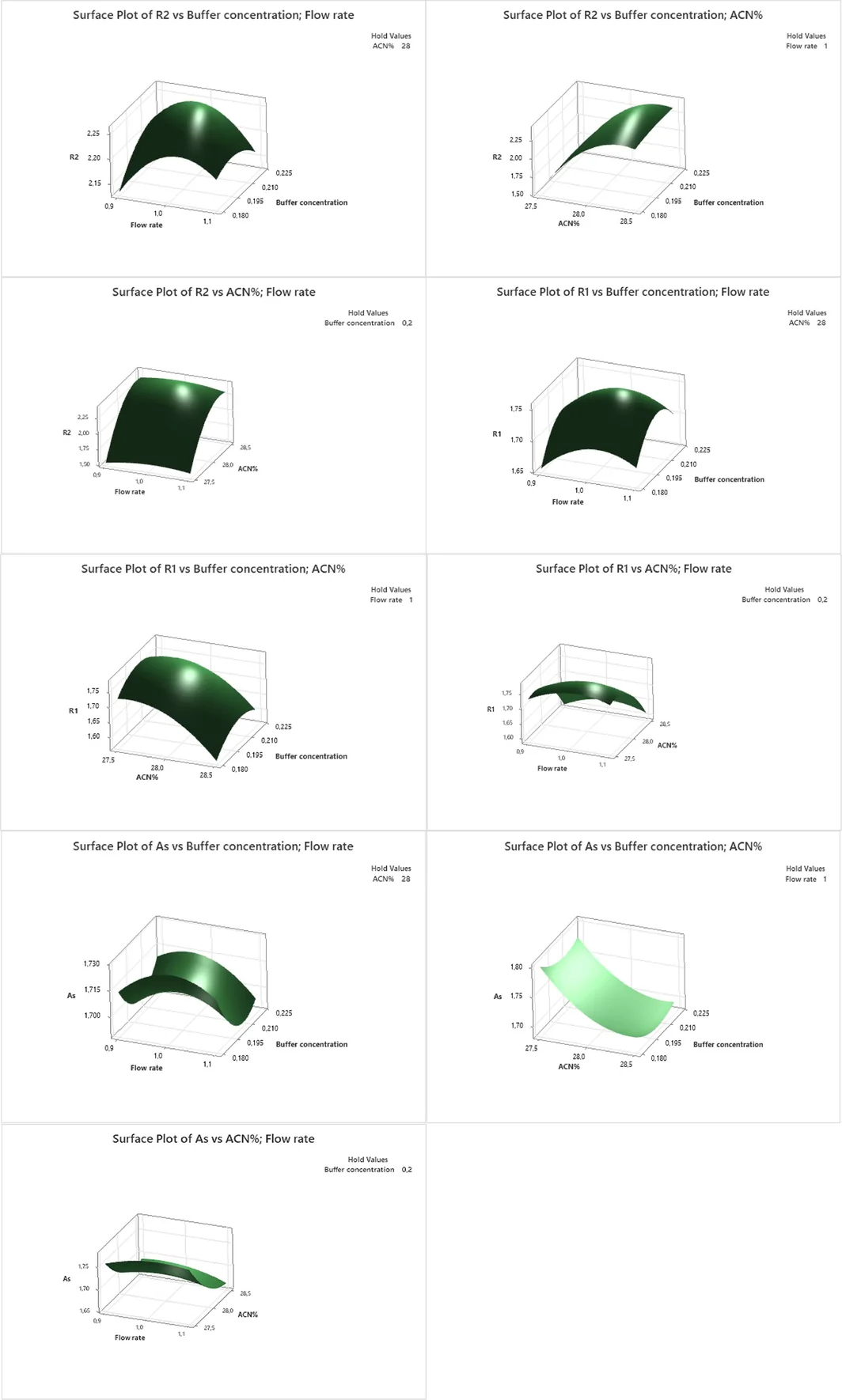
DoE surface plots.

##### 3.1.2.6 LOD and LOQ

The LOD and the LOQ were, respectively, 0.006 and 0.020 mg/mL (Figures 8 and 9).

**FIGURE 8:**
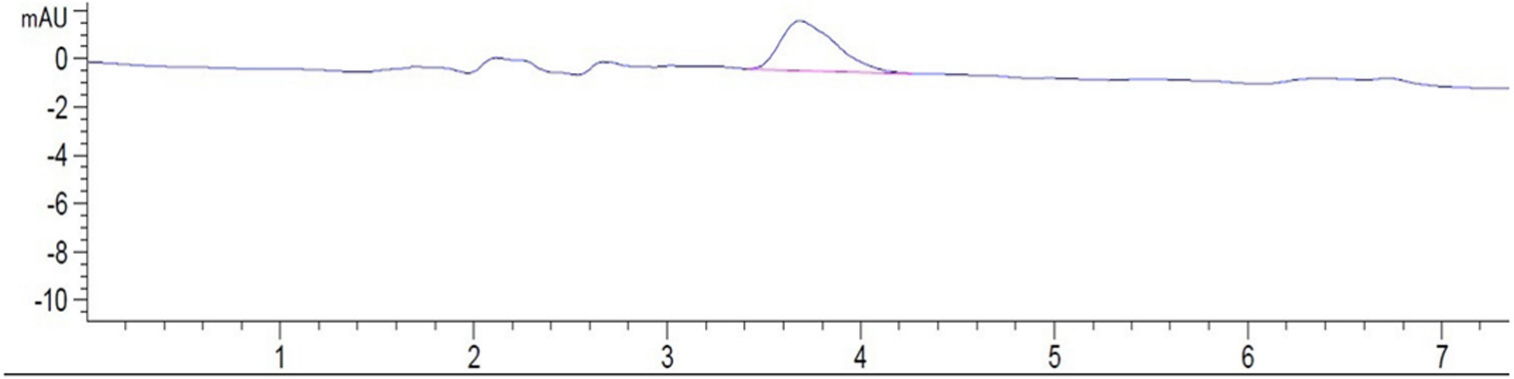
LOD chromatogram of insulin (0.006 mg/mL).

**FIGURE 9:**
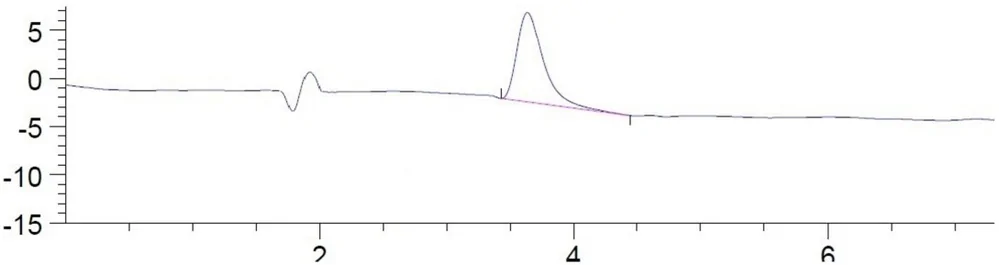
LOQ chromatogram of insulin (0.02 mg/mL).

#### 3.1.3 Control of Insulin Products and Stability Study

The results of the assay of insulin in the different marketed insulin products in Tunisia are summarized in Tables 3 and 4, respectively, at different time intervals including the first opening and after 10, 20, and 30 days at 25°C with pH ranging between 7.00 and 7.80 exposed to daylight and at pH ranging between 2.38 and 2.47 over 10, 20, and 30 days at 25°C exposed to daylight. During the stability study, the match factor for both insulin and A21‐desamido‐insulin peaks was 1000.

**TABLE 3:** Insulin control in pharmaceutical products (A and B) after the first opening and after 10, 20, and 30 days of exposure to daylight at 25°C.

Product	Insulin content at Day 0 (%)	Insulin content at Day 10 (%)	Insulin content at Day 20 (%)	Insulin content at Day 30 (%)
Insulin soluble A	99.89 (0.79)^a^	98.57 (0.95)^a^	98.15 (0.76)^a^	97.70 (0.89)^a^
Insulin soluble B	103.29 (0.84)^a^	98.56 (0.97)^a^	97.02 (0.82)^a^	96.98 (0.79)^a^
Insulin isophane biphasic A	105.00 (1.53)^a^	101.84 (0.78)^a^	103.02 (1.20)^a^	100.94 (0.85)^a^
Insulin isophane biphasic B	100.95 (1.43)^a^	100.75 (0.88)^a^	100.29 (0.88)^a^	100.12 (0.83)^a^
Insulin isophane A	102.88 (1.61)^a^	102.34 (0.68)^a^	102.10 (0.81)^a^	101.64 (0.89)^a^
Insulin isophane B	105.06 (0.90)^a^	104.91 (0.74)^a^	100.75 (0.87)^a^	99.57 (1.06)^a^

*Note:* A21‐desamido‐insulin was not detected.

^a^Standard deviation; *n* = 3; each preparation was repeated three times.

**TABLE 4:** Insulin control in pharmaceutical products (A and B) at diluted state (1 mg/mL in HCl 0.01 M) after 10, 20, and 30 days of exposure to daylight at 25°C.

Product	Insulin content at Day 0 (%)	Insulin content at Day 10 (%)	A21‐desamido‐insulin content at Day 10 (%)	Insulin content at Day 20 (%)	A21‐desamido‐insulin content at Day 20 (%)	Insulin content at Day 30 (%)	A21‐desamido‐insulin content at Day 30 (%)
Insulin soluble A	99.89 (0.79)^a^	85.38 (1.08)^a^	10.25 (0.93)^a^	77.63 (1.15)^a^	19.79 (0.78)^a^	75.81 (0.31)^a^	20.19 (1.57)^a^
Insulin soluble B	103.29 (0.84)^a^	86.80 (0.98)^a^	11.14 (1.14)^a^	85.29 (0.79)^a^	11.92 (1.52)^a^	84.30 (0.86)^a^	14.10 (0.81)^a^
Insulin isophane biphasic A	105.00 (1.53)^a^	95.98 (0.93)^a^	3.75 (0.58)^a^	89.14 (0.82)^a^	16.17 (1.49)^a^	87.86 (0.92)^a^	17.18 (1.34)^a^
Insulin isophane biphasic B	100.95 (1.43)^a^	100.43 (1.01)^a^	0.53 (0.12)^a^	89.94 (1.13)^a^	7.05 (0.71) ^a^	89.62 (1.05)^a^	11.29 (1.40)^a^
Insulin isophane A	102.88 (1.61)^a^	93.68 (0.73)^a^	3.62 (0.34)^a^	90.43 (0.77)^a^	12.13 (0.64)^a^	88.50 (0.86)^a^	12.10 (1.36)^a^
Insulin isophane B	105.06 (0.90)^a^	101.71 (0.84)^a^	1.42 (0.38) ^a^	96.45 (0.91)^a^	7.39 (1.06)^a^	91.07 (0.88)^a^	12.31 (0.70)^a^

*Note:* A21‐desamido‐insulin was detected starting from day 10.

^a^Standard deviation; *n* = 3, each preparation was injected three times.

### 3.2 Zinc Assay Method

#### 3.2.1 Method Validation

##### 3.2.1.1 Specificity

The average absorbance of six standard solutions containing zinc at 1 μg/mL in two different conditions, whether with and without the matrix, was, respectively, 0.0851 (± 0.0021), 0.0837 (± 0.0015). The *p*‐value of the Mann–Whitney test was 0.296 > 0.05, proving no significant difference.

##### 3.2.1.2 Linearity

Two calibration curves D3 and D4 were established, respectively, for the zinc standard solution and the zinc standard solution within the matrix in the range of 0.2–1.6 μg/mL. The coefficients of determination *R* ^2^ were 0.995 and 0.996, and the regression equations of the calibration curves were *Y* = 0.075x + 0.008 and *Y* = 0.073x + 0.007, respectively, for D3 and D4. There was no significant difference in terms of intercepts (calculated *T*‐value T _cal_: 0.513 < tabulated *T*‐value *T*_tab_(0.05; 26): 2.056) and slopes (*T*_cal_: 1.074 < *T*_tab_(0.05; 26): 2.056) between D3 and D4.

The results of the linearity statistical tests are summarized in Table 5.

**TABLE 5:** Results of the statistical tests for the linearity study (Zinc assay method).

Test	Tabulated value	Calculated value for D3 (zinc without matrix)	Calculated value for D4 (zinc within matrix)
Homogeneity of the variance: Cochran (C)	*C*_tab_(0.05; 5; 2): 0.68	0.24	0.46
Existence of a significant slope: Fischer (*F*1)	*F*1_tab_(0.05; 1; 13): 4.67	1358.82	1699.00
Validity of the regression line (*F*2)	*F*2_tab_(0.05; 3; 10): 3.71	2.86	3.67
Comparison of the intercept with 0	*T*_tab_(0.05; 13): 2.16	0.39	0.38

##### 3.2.1.3 Precision

The coefficients of intraday and interday variations were, respectively, 0.05% and 0.16%. The variance homogeneity Cochran test passed with calculated *C*‐value C_cal_ 0.502 < tabulated *C*‐value *C*_tab_(0.05; 3; 6): 0.677.

##### 3.2.1.4 Accuracy

The average recovery was 101.33%, and its interval of confidence was between 98.04% and 104.62%.

The intragroup variance homogeneity Cochran test passed with calculated *C*‐value C_cal_ 0.67 < tabulated *C*‐value *C*_tab_(0.05; 5; 2): 0.68. The validity of average Fisher test passed with calculated *F*‐value *F*_cal_ 2.57 < tabulated *F*‐value *F*_tab_(0.05; 4; 10): 3.48.

##### 3.2.1.5 LOD and LOQ

The LOD and the LOQ were, respectively, 0.005 and 0.015 μg/mL.

#### 3.2.2 Control of Zinc in Insulin Products and Stability Study

The results of the assay of zinc in the different insulin products marketed in Tunisia at different time intervals including the first opening (first group) and after 30 days at 25°C (second group) are summarized in Table 6.

**TABLE 6:** Zinc control in pharmaceutical products (A and B) after the first opening and after 30 days of exposure to daylight at 25°C.

Product	Zinc content (in μg/mL) after the first opening	Zinc content (in μg/mL) after 30 days
Insulin soluble A	14.74 (0.83)^a^	15.20 (0.68)^a^
Insulin soluble B	9.42 (2.31)^a^	8.65 (0.23)^a^
Insulin isophane biphasic A	22.36 (1.28)^a^	22.58 (0.91)^a^
Insulin isophane biphasic B	20.33 (1.64)^a^	20.25 (0.40)^a^
Insulin isophane A	25.15 (0.44)^a^	25.63 (1.12)^a^
Insulin isophane B	22.08 (0.68)^a^	18.36 (0.53)^a^

^a^Standard deviation; *n* = 5; each preparation was repeated five times.

The *p*‐value of the Mann–Whitney test used for the comparison of zinc content between the two groups was 0.873 > 0.05, proving no significant difference.

## 4 Discussion

### 4.1 Insulin Assay Method

#### 4.1.1 Method Development

In this study, we successfully developed an efficient analytical method for the assay of insulin that achieves a quick analysis time (≤ 10 min), an excellent separation (*R* ≥ 1.5) between closed peaks, and a satisfactory peak shape (peak symmetry between 0.8 and 1.8) after several preliminary experiments as follows:

At the beginning of the method development, the yielded insulin peak was broad with tailing when using a mobile phase composed of 26% acetonitrile, 12% methanol, and 62% phosphate buffer pH 3 (as shown in Figure 1). This issue of peak‐tailing is usually encountered with large molecules such as insulin. Besides, in these conditions, the method was judged unsuitable due to the longtime of analysis (RT = 21.4 min), with no separation between insulin and its degradation product. Therefore, we have tried to improve this issue by increasing acetonitrile % in the mobile phase. Going from 26% to 28% had made a considerable change in the chromatogram. In fact, the RT of insulin moved from 21.4 to 3.4 min with reversal in the elution order of insulin and its excipients (phenol and cresol peaks) as demonstrated in Figures 1 and 4. This change in the elution order could be attributed to the considerable variation in insulin RT resulting from minor fluctuation in acetonitrile concentration (2%). In contrast, the retention times of phenol and M‐cresol peaks did not show significant changes (less than 1.5 min) under the same conditions.

Afterwards, by rising acetonitrile percentage to 30% while maintaining the percentage of methanol at 12% did not change the insulin RT more than 1 min with no separation between insulin and A21‐desamido‐insulin peaks (as shown in Figure 3). Hence, we retained the mobile phase at 28% of acetonitrile to have the best resolution, respectively, between insulin and A21‐desamido‐insulin peaks and between A21‐desamido‐insulin and phenol peaks. Then, we opted to increase the concentration of phosphate buffer from 0.1 to 0.2 M, which had significantly increased the separation of A21‐desamido‐insulin from insulin and phenol (as shown in Figure 5).

The DoE results obtained in robustness study showed that a slight increase in acetonitrile % would improve the resolution between A21 esamido insulin and phenol (R2) and improve insulin peak symmetry but regress the resolution between insulin and A21‐desamido‐insulin (R1). A slight decrease in acetonitrile % would do the opposite effect.

The chosen chromatographic conditions were proved optimal using the DoE optimization solution shown in Table 7.

**TABLE 7:** DoE optimized factors values.

Solution	Flow rate	ACN%	Buffer concentration	As fit	R2 fit	R1 fit	Composite desirability
1	1.04343	28.1566	0.204242	1.68981	2.34023	1.72637	0.468290

The peak of A21‐desamido‐insulin was identified by injecting the corresponding solution of A21‐desamido‐insulin, prepared on the basis of some protocols reported in the literature. For example, the USP (2024) insulin monograph mentioned that 1.5 mg/mL of insulin solution in HCl 0.01 M kept at room temperature for 3 days could degrade 5% of insulin to A21‐desamido‐insulin. Also, Najjar et al. (2014) found that incubating an insulin solution at 1362 mg/mL in HCl 0.01 M for 4 days at 35°C may degrade all the insulin to A21‐desamido‐insulin. Moslemi et al. (2003) prepared A21‐desamido‐insulin by storing human insulin in 0.01 M of HCl at 40°C for 48 h. Thus, for our protocol, we had prepared the A21‐desamido‐insulin by incubating a 0.2mg/mL of standard insulin solution in HCl 0.01 M at 37°C. Then, we assessed the rate of insulin degradation by assay of this prepared solution every 7 days. After 28 days, insulin was degraded at 90%.

Concerning the developed method based on a gradient mode, the insulin and its degradation product were eluted after the phenol and M‐Cresol excipients (as demonstrated in Figure 6). The peak shape of insulin and A21‐desamido‐insulin was better than those obtained with the isocratic method, as we have got a symmetry factor under 1.7. Moreover, the separation between closed peaks was efficient through the increased resolution values. The run time for both the isocratic and the gradient methods was about 7 min.

Following literature, Najjar et al. (2014) used for the assay of insulin a RP‐C18 column (100 × 4.6 mm, 3‐μm particle) and a mobile phase consisting of a mixture of phosphate buffer 0.1 M, acetonitrile, and methanol, respectively, at 62%, 26%, and 12% (V/V/V), with a final pH of 3. The flow rate was 1 mL/min, and the detection was set at 214 nm. Under these conditions, the insulin peak was eluted at 7.87 min and the A21‐desamido‐insulin peak was eluted at 9.36 min. These two peaks were eluted after the excipients (phenol and cresol) and the internal standard (nitrophenol). Moslemi et al. (2003) developed a method for the assay of insulin by using a C18 column (300 × 3.9 mm, 10‐μm particle) with 3.9% tetra‐methyl‐ammonium hydroxide solution in water and acetonitrile (70:30, v/v) at pH 2.5 as mobile phase. The flow rate was set to 1 mL/min, and the detection was set at 214 nm. The retention times of insulin and the A21‐desamido‐insulin were, respectively, 16.76 and 19.99 min. These two peaks were eluted after excipients. By comparison with our developed method, we have got the advantage of a short time analysis since insulin and its degradation product, A21‐desamido‐insulin, were eluted, respectively, at 3.74 and 4.27 min with a total run time of 7 min.

Kaufmann and Boulle (2021) had adapted for the assay of insulin, a developed method based on a C18 column (100 × 2.1 mm, 3.5 μm, 100 A) maintained at 40°C, and two mobile phases, phase A (water +0.1% trifluoroacetic acid) and phase B (acetonitrile +0.1% trifluoroacetic acid), used in a gradient mode as follows: from 20% to 50% in 2 min for phase B, hold in 50% for 0.2 min, then decreased to initial conditions in 0.1 min. The flow rate was set at 0.5 mL/min, and the detection was set at 280 and 214 nm. By this method, the total run time was very rapid within 4 min that is well suitable for routine use. However, this method requires a binary pump, which is not always available in most laboratories. In addition, it does not make the separation of insulin from its degradation product, A21‐desamido‐insulin.

#### 4.1.2 Method Validation

Following the previous assays of optimization, we found that the isocratic method was simple, cost‐effective, and more achievable in routine analysis than the gradient method, which is laborious to be implemented. Therefore, the isocratic method had been selected for both validation and insulin stability study.

Our developed method was judged to be specific since no co‐eluting substances, and no interferences were detected in the peaks of insulin, A21‐desamido‐insulin, and excipients (phenol and cresol). The selected concentration range of insulin was higher than that cited by Najjar et al. (2014) (ranged from 0.01 to 0.68 mg/mL insulin) and Moslemi et al. (2003) (ranged from 0.01 to 0.1 mg/mL insulin).

For precision and accuracy criteria, all of the yielded results were within the acceptable limits. Indeed, the CV values for intraday and interday precision were between 0.61% and 0.63% (≤ 2%). The average recovery (*R* = 99.86%) was within the confidence interval (98.68%–101.04%). Therefore, our developed method by the HPLC‐UV is reliable, specific, accurate, and reproducible.

By comparison with other methods reported in literature, the study of Najjar et al. (2014) found the CV of repeatability at 0.91% and the mean recovery at 101.4%, while the study of Moslemi et al. (2003) found the CV of repeatability at 1.29%; the CV of reproducibility at 5.24% and the mean recovery at 97.35%. Hence, our method offers the highest precision than these two reported studies due to the lowest repeatability and reproducibility CV. Contrariwise, the method of Kaufmann and Boulle (2021) exhibits the best precision (CV of repeatability ≤ 0.23%).

Although the calculated values of LOD and LOQ were slightly higher than those reported in the other cited studies (Najjar et al. 2014; Moslemi et al. 2003), they are quite realistic for routine insulin analysis.

#### 4.1.3 Products Control and Insulin Stability Study

After the development and validation of an analytical method specific to the assay of insulin, we have applied it to the control of six insulin marketed products. Our obtained results were between 90% and 110% of the content of insulin as mentioned on the product label with no detection of A21‐desamido‐insulin, which complies with the limits established by the European pharmacopeia (2023). Concerning degradation products, A21‐desamido‐insulin peak area should not be greater than 5% of the total area of the peaks and the sum of the areas of any other peaks, apart from those due to insulin and A21‐desamido‐insulin should not be greater than 6% of the total area of the peaks disregard the peaks due to the excipients according to European pharmacopeia (2023). We did not detect A21‐desamido‐insulin peak or any other additional peak during the stability study of the insulin products.

Hence, we can conclude that insulin in the products at pH ranging between 7 and 7.8 is well stable when exposed to daylight at 25°C for at least 30 days. Following literature, some studies had only revealed that insulin may be stable at high temperature without testing the effect of the daylight exposure (Kaufmann and Boulle 2021). For example, the study of Kaufmann et al. found that insulin products kept at oscillating temperatures between 25°C and 37°C were stable for 12 weeks. These oscillating temperatures were recorded at Dagahaley refugee camp in Northern Kenya, where access to refrigeration was difficult for patients.

Moses et al. (2019) had also assessed the impact of several factors such as storage longevity, cold chain transport, pharmacy handling, and in‐use conditions on the concentration of insulin whether in its regular or isophane form in several marketed products. They found that insulin was stable after stressed conditions: full shelf life storage for 30 months at 5°C, cold chain transport, and pharmacy handling as the recommended procedure (5°C ± 3°C) with additional four weeks of storage at 30°C. Hence, following these results insulin was recommended to be stored for 6 weeks at 30°C to mimic in‐use impact.

Baechler et al. (2020) had also assessed the stability of five different insulin products over 3 months at 4°C, 22°C, and 37°C, using high‐resolution mass‐spectrometry. As a result, they did not found any chemical instability as the measured insulin content remains in accordance with the FDA and EMA requirements.

Contrariwise, to the above detected results, the insulin products stored in diluted form at 1 mg/mL in HCl 0.01 M with pH ranging between 2.38 and 2.47 were found less stable, due to the appearance of the insulin degradation product, A21‐desamido‐insulin in the chromatogram (as shown in Figure 10). In fact, after 10 days at 25°C, insulin content dropped about 4% or more in all products except for Insulin isophane biphasic B which dropped slightly. This could be explained by the specific formulation containing protamine–zinc–insulin complex since all protamine including insulin products dropped less than regular insulin. In fact, the crystalline NPH fraction exhibits reduced molecular mobility and lower susceptibility to chemical degradation pathways such as desamidation, oxidation, and aggregation. Also, the specific preservatives proportions could further contribute to maintaining insulin integrity. Nevertheless, in all cases, the stability decreases within time, and the diluted forms of insulin are less stable than the nondiluted forms. After 30 days, all products were out of specification: Five had insulin content below 90% and one had insulin content over 90%, but A21‐desamido‐insulin was very high (12.31%).

**FIGURE 10:**
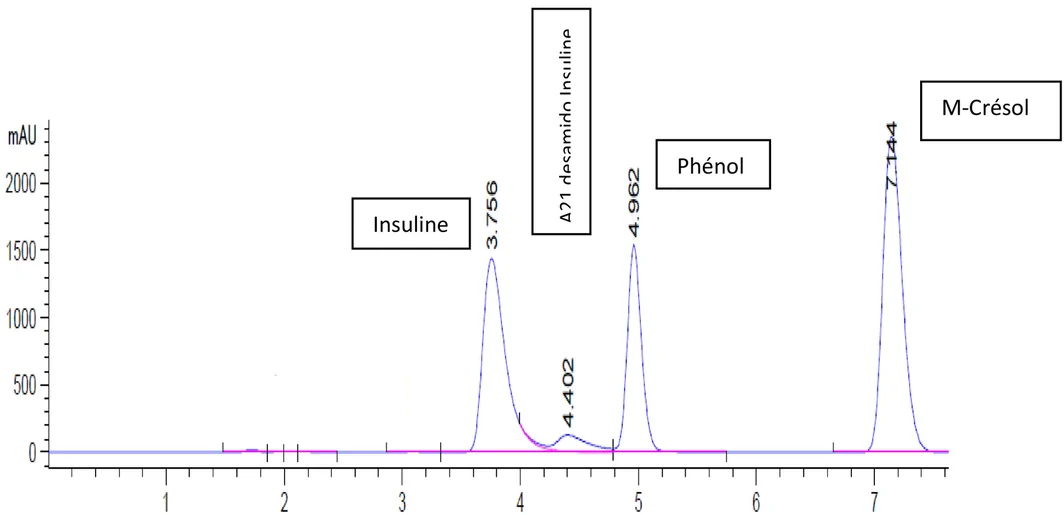
Chromatogram of insulin isophane (B) at pH 2 after 30 days of exposure to daylight at 25°C.

Similarly, the study of Moslemi et al. (2003) had also tested the stability of diluted forms of insulin at pH 2, respectively, for 12, 24, and 72 h at 25°C, finding that after 24 h, there was no significant degradation of insulin. The study of Najjar et al. had also revealed that insulin was instable after 12 h at room temperature when diluted in an acidic medium. This instability is probably due to the effect of pH more than the incriminated role of dilution. Indeed, insulin was found to be stable at pH between 5 and 8 (Brange and Langkjaer 1992). Moreover, Mohr et al. (2021) had also tested the stability of insulin solution diluted at 1 UI/mL (34.7 μg/mL) in 0.9% NaCl with pH above 6.5, revealing that the stability was up to 21 days in dark at 2°C–8°C and up to 48 h at 20°C–25°C when exposed to daylight, which confirms as well the influence of pH on the stability of insulin.

### 4.2 Zinc Assay Method

#### 4.2.1 Method Validation

Our developed method for the assay of zinc is judged to be selective. In fact, the two absorbance values of the zinc solution at 1 μg/mL, whether with or without the presence of matrix, were comparable as the *p*‐value of the Mann–Whitney test was superior to 0.05, which means no significant difference between the two groups.

Following literature, our selected range concentration (0.2–1.6 μg/mL) exceeds the range selected by Ata et al. (2015), which was between 0.2 mg/L and 1.2 μg/mL. For the study of Tănase et al. (2006), the range was between 0.05 and 1 μg/mL, and for the study of Shkunnikova et al. (2021), the range was between 0.05 and 1.2 μg/mL. Accordingly, all the reported ranges were close to each other.

For the precision and accuracy criteria, all the yielded results fulfilled the acceptance criteria. The intraday and interday precision CV values were between 0.05% and 0.16% (≤ 2%). For the accuracy, the average recovery (*R* = 101.33%) was within the confidence interval [98.04%–104.62%].

Therefore, our study proves well that the AAS method is reliable, accurate and reproducible. By referring to the other reported studies, Ata et al. (2015), by using a Hitachi SAA model A‐1800, found that the coefficients of variation for repeatability and reproducibility were, respectively, 0.18% and 0.47%. The mean recovery was 101%, which is close to our result. Similarly, Tănase et al. (2006), by using an SAA SOLAAR 32 AA, found the coefficients of variation for repeatability and reproducibility, respectively, at 2.37% and 2.69%, and the mean recovery was 99.73%. Shkunnikova et al. (2021), by using AAnalyst 800 SAA (Perkin Elmer) found the CV for repeatability between 1.11% and 2.52% and the mean recoveries between 95.6% and 100.1%.

Hence, our method exhibits the lowest values for the repeatability and reproducibility CVs when compared to those reported methods, while the mean recovery was closer to that of Ata et al. (2015). Although the LOD and LOQ were slightly higher than those reported in other methods (Shkunnikova et al. 2021; Ata et al. 2015; Tănase et al. 2006), they are quite suitable for assay of zinc in insulin products.

#### 4.2.2 Products Control and Zinc Stability Study

According to the European Pharmacopoeia (2023) and the USP (2024), the uppermost permissible threshold for zinc in human insulin products is 40 μg/mL. Following our control study, we revealed that all the Tunisian marketed human insulin products have zinc concentrations below 40 μg/mL; hence, they comply with the European and US pharmacopeias.

The *p*‐value of the Mann–Whitney test was superior to 0.05, proving that there was no significant difference in the zinc content at the initial time of vials opening and after storage for 30 days at 25°C. Hence, zinc remains stable in the Tunisian marketed human insulin products over 30 days at 25°C exposed to daylight.

### 4.3 Comparison Between HPLC‐UV and AAS Methods in Terms of Their Effectiveness

The developed method by the AAS is judged to be very simple and rapid since there are few analytical parameters to be optimized such as gas mixture and gas flow. Contrariwise, the development of a new method by HPLC‐UV approach is more challenging since there are many analytical parameters to be optimized such as mobile phase composition, pH, buffer nature and concentration, type of the stationary phase, flow rate, elution mode (isocratic, gradient), column temperature, and detector wavelength.

In addition, method validation could be influenced by the type of equipment. By way of illustration, when using the AAS method, the hollow cathode lamp allows the specific determination of the analyzed element. However, by this method, interferences coming from other elements absorbing at wavelengths close to the analyzed element must be checked. For the HPLC‐UV approach, specificity criterion requires separation of the analyte from the various matrix constituents along with any degradation or other organic impurities, which is a time‐consuming process involving several reagents.

Both techniques have shown satisfactory linearity. The HPLC‐UV approach exhibits the higher coefficient of determination *R* ^2^, but the concentration range was about 1000 times greater than the AAS method.

The accuracy for both methods is acceptable. The AAS approach provides improved precision with lower CVs of repeatability and reproducibility. In addition, it displays a lower LOD and a LOQ than the HPLC approach. Strikingly, in routine analysis, it is a very rapid method allowing analysis within 1 min. However, the sole limitation by this approach is that only one element could be analyzed. Contrariwise, for the HPLC‐UV approach, although it displays a longer run time analysis, higher energy, and solvent consumption, specifically those of hazardous type, for example, methanol and acetonitrile, it has the advantage of analyzing multi analytes in only one single run. In addition, it allows performing the stability study of pharmaceutical products for the detection and quantification of degradation products. The only limited issue by this approach is that it requires at the beginning of each analysis, approximately 1 h to adjust the baseline and reach the equilibrium of the stationary phase. Then, at the end of work, column and capillaries should be washed, mainly after running a buffer in the mobile phase, when compared to the AAS approach that only requires the injector washing within only few seconds.

Overall, we can resume that each of the HPLC‐UV and AAS methods exhibits some advantages and disadvantages; that is, why combining both methods is frequently adapted to meet the regulatory quality control requirements.

## 5 Conclusion

We succeeded in our study in the development of two analytical methods based on the HPLC‐UV and AAS approaches, respectively, for the assay of insulin and Zinc. The both developed methods were judged to be simple, rapid, selective, precise, and accurate, following the ICH requirements, via providing precise measurements in a very straightforward and timely manner. Consequently, they could be adapted for routine analysis of pharmaceutical product‐based insulin and zinc, as we have confirmed in our study. The results of our stress‐degradation stability study, when controlling some of the Tunisian marketed pharmaceutical product‐based insulin, revealed a good stability of insulin when exposed to daylight for up to 30 days at 25°C, neutral pH in its primary package. However, in diluted acidic medium (HCl 0.01 M, pH 2), the risk of degradation is higher. Therefore, freshly prepared solutions should be made for each control of insulin by HPLC analysis.

In summary, our study has enabled us to establish some stability conditions for the pharmaceutical product‐based insulin, paving the way for other investigations to complete the long‐term stability study for an extended duration at 25°C under daylight exposure.

Additionally, other studies are warranted in order to assess the stability of insulin products if the cold chain is not respected in hot tropical regions where temperatures exceed 37°C, in order to define the practical insulin stability conditions and eventually changing the storage condition recommendations to help patients with scarce finances or living in remote areas.

## Author Contributions

**Majdi Ben Messaoud:** research/formal analysis/writing. **Sarra Mahfoudhi:** conceptualization/supervision. **Dorra Amor:** supervision/equipment supply. **Yassine Mokni:** formal analysis. **Kaouther Louati:** revision. **Kaouther Zribi:** supervision. **Asma Omezzine:** supervision. **Fathi Safta:** supervision/revision/correction/project administration.

## Conflicts of Interest

The authors declare no conflicts of interest.

## Data Availability

The data that support the findings of this study are available from the corresponding author upon reasonable request.

## References

[bmc70585-bib-0001] Ata, S. , F. H. Wattoo , M. Ahmed , M. H. S. Wattoo , S. A. Tirmizi , and A. Wadood . 2015. “A Method Optimization Study for Atomic Absorption Spectrophotometric Determination of Total Zinc in Insulin Using Direct Aspiration Technique.” Alexandria Journal of Medicine 51, no. 1: 19–23. 10.1016/j.ajme.2014.03.004.

[bmc70585-bib-0002] Baechler, F. , C. Stettler , B. Vogt , L. Bally , and M. Groessl . 2020. “Concentration and Chemical Stability of Commercially Available Insulins: A High‐Resolution Mass Spectrometry Study.” Diabetes Technology & Therapeutics 22, no. 4: 326–329. 10.1089/dia.2019.0412. 32031881

[bmc70585-bib-0027] Banting, F. G. , and C. H. Best . 1987. “The Internal Secretion of the Pancreas.” Journal of Laboratory and Clinical Medicine 45, no. 2: 7–55. 10.1111/j.1753-4887.1987.tb07442.x 3550540

[bmc70585-bib-0004] Brange, J. , and L. Langkjaer . 1992. “Chemical Stability of Insulin. 3. Influence of Excipients, Formulation, and pH.” Acta Pharmaceutica Nordica 4, no. 3: 149–158. 1418641

[bmc70585-bib-0005] Dunn, M. F. 2005. “Zinc‐Ligand Interactions Modulate Assembly and Stability of the Insulin Hexamer: A Review.” Biometals 18, no. 4: 295–303. 10.1007/s10534-005-3685-y. 16158220

[bmc70585-bib-0006] European Pharmacopoeia . 2023. “Insulin Preparations, Injectable (Monograph 0854).” Strasbourg, France: Council of Europe.

[bmc70585-bib-0007] Fisher, A. M. 1955. “Insulin Preparations.” Canadian Medical Association Journal 73, no. 1: 1–8. PMC1825981 14390036

[bmc70585-bib-0008] Hall, A. G. , and J. C. King . 2022. “Comparison of Serum, Plasma, and Liver Zinc Measurements by AAS, ICP‐OES, and ICP‐MS in Diverse Laboratory Settings.” Biological Trace Element Research 200, no. 6: 2606–2613. 10.1007/s12011-021-02883-z. PMC9132797 34453311

[bmc70585-bib-0009] Heinemann, L. , K. Braune , A. Carter , A. Zayani , and L. A. Krämer . 2021. “Insulin Storage: A Critical Reappraisal.” Journal of Diabetes Science and Technology 15, no. 1: 147–159. 10.1177/1932296819900258. PMC7783014 31994414

[bmc70585-bib-0010] International Council for Harmonisation of Technical Requirements for Pharmaceuticals for Human Use . 2023. “ICH Q2(R2) Validation of Analytical Procedures. European Medicines Agency.”

[bmc70585-bib-0011] Kaufmann, B. , and P. Boulle . 2021. “Heat‐Stability Study of Various Insulin Types in Tropical Temperature Conditions: New Insights Towards Improving Diabetes Care.” PLoS ONE 16, no. 2: e0245372. 10.1371/journal.pone.0245372. PMC7857579 33534816

[bmc70585-bib-0012] Marukhlenko, A. , T. Maksimova , T. Pleteneva , and M. Morozova . 2021. “Development and Validation of a Method for the Quantitative Determination of Zinc in Its Chelate Complexes Using Energy Dispersive X‐Ray Fluorescence Spectroscopy.” Drug Development & Registration 10: 154–161. 10.33380/2305-2066-2021-10-4-154-161.

[bmc70585-bib-0013] Mayer, J. P. , F. Zhang , and R. D. DiMarchi . 2007. “Insulin Structure and Function.” Biopolymers 88, no. 5: 687–713. 10.1002/bip.20734. 17410596

[bmc70585-bib-0014] Mohr, A. , F. Erdnüß , and I. Krämer . 2021. “Physicochemical Stability of Human Insulin 1 I.U./mL Infusion Solution in 50 mL Polypropylene Syringes.” Pharmaceutical Technology in Hospital Pharmacy 6: 20210005. 10.1515/pthp-2021-0005.

[bmc70585-bib-0015] Moses, A. , J. Bjerrum , M. Hach , L. H. Wæhrens , and A. D. Toft . 2019. “Concentrations of Intact Insulin Concur With FDA and EMA Standards When Measured by HPLC in Different Parts of the Distribution Cold Chain.” Journal of Diabetes Science and Technology 13, no. 1: 55–59. 10.1177/1932296818783783. PMC6313285 29952654

[bmc70585-bib-0016] Moslemi, P. , A. R. Najafabadi , and H. Tajerzadeh . 2003. “A Rapid and Sensitive Method for Simultaneous Determination of Insulin and A21‐Desamido Insulin by High‐Performance Liquid Chromatography.” Journal of Pharmaceutical and Biomedical Analysis 33, no. 1: 45–51. 10.1016/S0731-7085(03)00336-4. 12946530

[bmc70585-bib-0017] Najjar, A. , M. Alawi , N. Abu Heshmeh , and A. Sallam . 2014. “A Rapid, Isocratic HPLC Method for Determination of Insulin and Its Degradation Product.” Advances in Pharmaceutics 2014: 1–6. 10.1155/2014/749823.

[bmc70585-bib-0018] Scheen, A. , and P. Lefèbvre . 2021. “L'épopée des insulines des années 1930 aux années 1980.” Médecine Des Maladies Métaboliques 2557, no. 21: 3S25–3S22. 10.1016/S1957-2557(21)00177-2.

[bmc70585-bib-0019] Shen, Y. , W. Prinyawiwatkul , and Z. Xu . 2019. “Insulin: A Review of Analytical Methods.” Analyst 144: 1–14. 10.1039/C9AN00112C. 31143899

[bmc70585-bib-0020] Shkunnikova, S. Š. , K. Lalić , A. Kulišić , and J. Jablan . 2021. “Comparison of Zn Content in Rapid‐Acting Insulin and Biphasic Suspension by FAAS.” Croatica Chemica Acta 94, no. 2: 1–8. 10.5562/cca3830.

[bmc70585-bib-0021] Shvetsova, Y. N. , A. A. Erokhina , E. A. Khorolskaya , E. S. Zhigilei , and N. E. Kuz'mina . 2023. “Analytical Method Development and Validation for Insulin and Zinc Control.” Regulatory Research and Medicine Evaluation 13, no. 3: 394–402.

[bmc70585-bib-0022] Song, M. K. , N. F. Adham , and H. Rinderknecht . 1976. “A Simple, Highly Sensitive Colorimetric Method for the Determination of Zinc in Serum.” American Journal of Clinical Pathology 65, no. 2: 229–233. 10.1093/ajcp/65.2.229. 766608

[bmc70585-bib-0023] Tănase, I. G. , A. Pana , and A. Pană . 2006. “An Analytical Method Validation for Atomic Absorption Spectrometry Analysis of Total Zinc From Insulin.” Chimie (Serie Noua) 15, no. 2: 7–14.

[bmc70585-bib-0024] United States Pharmacopeial Convention . 2024. “Insulin.” In USP–NF 47.

[bmc70585-bib-0025] Vanga, N. R. , V. R. Kesamsetty , and K. Ummiti . 2024. “Development and Validation of a Method for Trace Level Zinc Quantification in Pharmaceutical Zinc Supplements Using a Carboxyl Functional Group Packed Column and Refractive Index Detector.” Journal of AOAC International 107, no. 1: 2–13. 10.1093/jaoacint/qsad101. 37676824

